# Caminho na rede formal de cuidado em saúde de pacientes pós-alta hospitalar segundo multimorbidade**

**DOI:** 10.15649/cuidarte.1279

**Published:** 2022-08-07

**Authors:** Melina Lopes Lima, Danielle Lopes Bordin, Renata Cristini Fernandes Furquim, Luciane Patrícia Andreani Cabral, Erildo Vicente Muller, Cristina Berger Fadel

**Affiliations:** 1 Universidade Estadual de Ponta Grossa. Ponta Grossa-PR, Brasil. E-mail: enfmelina@gmail.com Universidade Estadual de Ponta Grossa Universidade Estadual de Ponta Grossa Ponta Grossa PR Brazil enfmelina@gmail.com; 2 Departamento de Enfermagem e Saúde Pública da Universidade Estadual de Ponta Grossa (UEPG), Ponta Grossa PR, Brasil. E-mail: daniellebordin@hotmail.com Universidade Estadual de Ponta Grossa Departamento de Enfermagem e Saúde Pública Universidade Estadual de Ponta Grossa Ponta Grossa PR Brazil daniellebordin@hotmail.com; 3 Hospital Universitário Regional dos Campos Gerais. Ponta Grossa-PR, Brasil. E-mail: renata_furquim@hotmail.com Hospital Universitário Regional dos Campos Gerais Ponta Grossa PR Brasil renata_furquim@hotmail.com; 4 Hospital Universitário Regional dos Campos Gerais. Ponta Grossa-PR, Brasil. E-mail: luciane.pacabral@gmail.com Hospital Universitário Regional dos Campos Gerais Ponta Grossa PR Brasil luciane.pacabral@gmail.com; 5 Departamento de Enfermagem e Saúde Pública da Universidade Estadual de Ponta Grossa (UEPG), Ponta Grossa PR, Brasil. E-mail:erildomuller@uepg.br Universidade Estadual de Ponta Grossa Departamento de Enfermagem e Saúde Pública Universidade Estadual de Ponta Grossa Ponta Grossa PR Brazil erildomuller@uepg.br; 6 Departamento de Odontologia da Universidade Estadual de Ponta Grossa (UEPG), Ponta Grossa-PR, Brasil. E-mail: cbfadel@gmail.com Universidade Estadual de Ponta Grossa Departamento de Odontologia Universidade Estadual de Ponta Grossa Ponta Grossa PR Brazil cbfadel@gmail.com

**Keywords:** Assistência Centrada no Paciente, Multimorbidade, Hospitalização, Assistência à Saúde, Sistema Único de Saúde, Patient-Centered Care, Multimorbidity, Hospitalization, Delivery of Health Care, Unified Health System, Atención Dirigida al Paciente, Multimorbilidad, Hospitalización, Prestación de Atención de Salud, Sistema Único de Salud

## Abstract

**Introdução:**

A gestão de informações associadas à multimorbidade na atenção hospitalar é relevante para o planejamento de estratégias de prevenção de agravos à saúde em pacientes de maior risco, a fim de oportunizar a organização de sistemas de saúde de modo eficiente.

**Objetivo:**

O presente trabalho objetivoudelinear o caminho formal percorrido por pacientes com e sem multimorbidade, considerando o uso da rede pública de saúde após a internação hospitalar.

**Materiais e métodos:**

Realizou-se um estudo quantitativo, transversal, descritivo, utilizando dados primários de 445 pacientes internados em um hospital universitário, no ano de 2018. Os dados foram coletados através de análise do prontuário médico e entrevista telefônica. Os resultados foram analisados por meio de frequência absoluta e relativa.

**Resultados:**

Desenvolveu-se um fluxograma, representando os pontos da rede de saúde utilizados pelo paciente após a alta hospitalar, segundo multimorbidade. Verificou-se uma alta prevalência de encaminhamento (com multimorbidade (CM) 93,52%; sem multimorbidade (SM) 97,71%) e comparecimento na atenção secundária à saúde (CM 86,15%; SM 89,63%), um baixo encaminhamento (CM 42,45%; SM 36,27%) e comparecimento na atenção primária à saúde (CM 61,29%; SM 64,81%), e considerando os 3 níveis de atenção juntos, houve um baixo comparecimento (CM 17,98%; SM 21,89%) para ambos os grupos investigados.

**Discussão:**

Entende-se que a semelhança de comparecimento em todos os pontos da rede por ambos os grupos é um problema, por tratar de forma igual populações desiguais e consequentemente com necessidades diversas.

**Conclusões:**

Sinaliza-se a importância de maior incentivo ao acompanhamento de pacientes com multimorbidade na rede primária de saúde, especialmente no período de pós-alta hospitalar, e a necessidade de fortalecimento da Rede de Atenção à Saúde.

## Abstract (en)

**Introduction:**

Managing information related to multimorbidity in hospital care is crucial for planning strategies to prevent health problems in high-risk patients in order to enable healthcare systems to be organized more efficiently.

**Objective:**

To describe the formal healthcare network path for patients with and without multimorbidity in relation to the use of public healthcare networks after discharge.

**Materials and Methods:**

A quantitative descriptive cross-sectional study was conducted with primary data from 445 patients admitted to a university hospital in 2018. Data were collected through analysis of medical records and telephone interviews. Results were analyzed by absolute and relative frequency.

**Results:**

A flowchart was developed representing the healthcare network locations used by the patient after hospital discharge based on morbidity. It was possible to confirm the existence of a high prevalence of referral (with multimorbidity (WM): 93.52%, without multimorbidity (WOM): 97.71%) and secondary care attendance, low prevalence of referral (WM: 42.45%, WOM: 36.27%) and primary care attendance (WM: 61.29%, WOM: 64.81%). When putting the three levels of healthcare together, low attendance (WM: 17.98% WOM: 21.89%) was observed in both groups under study.

**Discussion:**

Similar attendance at all healthcare network locations is problematic as these are unequal populations and thus, with different needs

**Conclusions:**

It is important to encourage follow-up of patients with multimorbidity in the primary care network, especially in the period after hospital discharge and strengthen the healthcare network.

## Resumen

**Introducción:**

La gestión de la información asociada a la multimorbilidad en la atención hospitalaria es crucial para la planificación de estrategias de prevención de problemas de salud en los pacientes de mayor riesgo, con el fin de permitir organizar los sistemas de salud de forma eficiente.

**Objetivo:**

Describir la ruta formal recorrida por los pacientes con y sin multimorbilidades con base en el uso de la red pública de salud después de la hospitalización.

**Materiales y métodos:**

Se realizó un estudio cuantitativo, transversal y descriptivo con datos primarios de 445 pacientes ingresados en un hospital universitario en 2018. Los datos se recolectaron mediante el análisis de historias clínicas y entrevistas telefónicas. Los resultados se analizaron a través de frecuencia absoluta y relativa.

**Resultados:**

Se elaboró un flujograma que representa los puntos de la red de atención en salud utilizados por el paciente tras el alta hospitalaria según la morbilidad. Se confirmó la existencia de una alta prevalencia de remisión (con multimorbilidad (CM): 93.52%, sin multimorbilidad (SM): 97.71%) y asistencia a atención secundaria, baja remisión (CM: 42.45% SM: 36.27%) y asistencia a atención primaria (CM: 61.29% SM: 64.81%); al incluir los tres niveles de atención en conjunto se evidenció una baja asistencia (CM: 17.98% SM: 21.89%) en ambos grupos investigados.

**Discusión:**

Se considera problemático la similitud en la asistencia en todos los puntos de la red por parte de ambos grupos, por tratarse de poblaciones desiguales y, en consecuencia, con necesidades diferentes.

**Conclusiones:**

Se señala la importancia de incentivar el seguimiento de los pacientes con multimorbilidades en la red de atención primaria, especialmente en el periodo posterior al alta hospitalaria y la necesidad de reforzar la red de atención en salud.

## Introdução

A multimorbidade foi descrita pela primeira vez em 1976, na Alemanha, cuja definição é a ocorrência de duas ou mais doenças crônicas em um mesmo indivíduo^1^^,^^2^, com ausência de condição primária ou problema de saúde central^3^. Estudos demonstram prevalências de multimorbidade mundiais estimadas entre 12,9% e 95,1%^4^ e com variações atribuídas ao grupo etário e à discrepância na conceituação do fenômeno, sendo maior em idosos, mulheres^3^, estratos sociais com maior privação de renda^5^, pessoas com menor escolaridade^6^ e moradores de área urbana^7^.

A multimorbidade vem aumentando globalmente devido a vários fatores, dentre eles o envelhecimento populacional, o aumento de massa corporal, a urbanização e o aumento das condições crônicas não transmissíveis^3^. Quando se compara indivíduos com uma única condição crônica e aqueles multimórbidos, observa-se maior número de hospitalizações^8^, maior tempo de internação, uso do sistema de saúde e gasto com saúde mais elevados, mortalidade precoce^9^, capacidade funcional reduzida^5^, menor qualidade de vida^10^, perda de autonomia e maior fragilidade entre multimórbidos^6^.

Como a maioria das diretrizes e sistemas de saúde são organizados para tratar doenças isoladas^11^ e a formação em saúde no Brasil ser ainda excessivamente especializada^12^, é frequente que pacientes com multimorbidade sejam tratados por diversos profissionais de saúde,o que ocasiona um desvio no atendimento ideal, impossibilitando uma visão integral do processo saúde-doença. Nestes casos, é comum que alguns não consigam aderir a todas as designações médicas, devido à enorme demanda do tratamento de doenças isoladas^13^, podendo ainda, as consultas múltiplas, aumentar o risco de polifarmácia, decorrente da falha de comunicação entre os diferentes profissionais^3^, e levar à provável cascata de prescrições, acarretando iatrogenias ao portador de multimorbidade^5^, levando a um cuidado de saúde subótimo^3^ e à insatisfação do paciente^14^.

As evidências mostram que existem interações entre doenças e tratamentos, e entre questões biomédicas e de contexto social, tornando imprudente cuidar de cada condição separadamente, como o que acontece com o trabalho descoordenado de especialistas^13^. Nesse sentido, a Atenção Primária à Saúde (APS) representa uma das estratégias mais promissoras para o cuidado destes indivíduos devido ao seu papel de ordenação e integração de cuidados físicos, mentais e sociais, sendo ainda capaz de potencializar a centralidade do sujeito no cuidado à saúde.

Destaca-se aqui a APS também como ordenadora do cuidado da Rede de Atenção à Saúde (RAS) de pessoas com doenças crônicas, por ser o ponto da atenção com maior capilaridade e potencial para identificar as necessidades de saúde da população e capacidade de realizar a estratificação de riscos à saúde, a qual subsidiará toda a organização do cuidado em rede^15^. Assim, o funcionamento profícuo da RAS perpassa e é codependente do papel central da APS, por meio do trabalho compartilhado entre os seus profissionais e os especialistas focais.

O papel dos pontos de atenção ambulatorial especializada e de atenção hospitalar no cuidado às pessoas com doenças crônicas deve ser complementar e integrado à APS, superando a atuação fragmentada e isolada culturalmente arraigada tanto na formação dos profissionais de saúde quanto nos serviços de saúde no Brasil. Para tanto, se faz necessária que a oferta de serviços pela atenção secundária e terciária seja planejada a partir do ordenamento da RAS pela APS, e que o cuidado seja coordenado pelos profissionais da atenção primária^15^.

Dentro deste contexto e do conhecimento de que a multimorbidade é frequentemente excluída das investigações em saúde (pela complexidade de sua natureza e de seus desdobramentos), acredita-se na importância de compreender o caminho formal percorrido por pacientes com multimorbidade na rede pública de saúde, a fim de oportunizar a organização de sistemas de saúde de modo eficiente, eficaz e efetivo para o subsídio de estratégias de melhoria de atenção à saúde e de prevenção de complicações a pacientes de maior risco (subsidiando a elaboração de diretrizes de tratamento e o treinamento de profissionais de saúde, diminuindo o risco de hospitalizações evitáveis e de iatrogenias). Assim, idealizou-se o presente estudo que tem por objetivo delinear o caminho formal percorrido por pacientes com e sem multimorbidade, considerando o uso da rede pública de saúde após a internação hospitalar.

## Materiais e Métodos

### Tipo de Estudo

Trata-se de um estudo quantitativo, transversal, de caráter descritivo e inferencial, com utilização de dados primários obtidos por meio de entrevista telefônica junto a pacientes que estiveram internados, cuidadores ou familiares, no período de fevereiro a julho de 2018.

### População e Amostra

O local onde foi realizado o estudo é um hospital universitário, que está localizado no estado do Paraná, Brasil, sendo referência para doze municípios e aproximadamente 637 mil habitantes^16^.

O cálculo do tamanho amostral foi determinado através do software Epi. Info 7.1.4. Para tanto, considerou-se o valor médio mensal de pacientes internados no sistema de prontuário eletrônico do serviço (n=506,6) multiplicado por 6 (número de meses estimado para a coleta), resultando em uma amostra de 3.040 indivíduos. Para cálculo ponderou-se uma precisão de 5%, intervalo de confiança de 95% e efeito de desenho 1, para uma prevalência de 50% de adultos internados com multimorbidade. Utilizou-se esta prevalência na intenção de obtenção da maior amostra possível. Ao total calculado (n=342) foram acrescidos 103 indivíduos (30%), considerando as possíveis perdas, resultando na amostra final de 445 indivíduos.

Os critérios de elegibilidade foram: pacientes que permaneceram internados no hospital; membro familiar ou cuidador que tenha acompanhado integralmente o processo de internamento (quando o próprio indivíduo não apresentava condições de responder ao questionário); ter recebido alta hospitalar no mínimo 30 dias antes da realização da entrevista; tempo de internação superior a um dia, ser maior de 18 anos. Os critérios de exclusão foram: gestantes; pacientes com vírus da imunodeficiência humana (HIV) e hepatites virais(Esta população foi desconsiderada no estudo, pois na cidade de investigação estes pacientes são referenciados para uma rede própria); pacientes que vieram a óbito; pacientes que tiveram a cirurgia cancelada; pacientes que ficaram internados no Pronto Atendimento; pacientes que não apresentaram contato telefônico; pacientes que não atenderam ao telefone após três tentativas em dias e horários diferentes e pacientes, familiares ou cuidadores que não consentiram em participar do estudo.

### Coleta de Dados

Para a obtenção das informações dos pacientes desenvolveu-se um questionário estruturado, baseado em instrumentos propostos pelo Ministério da Saúde brasileiro^15^^,^^17^e publicações^18^^,^^19^^,^^20^, contendo questões que validassem a presença das doenças crônicas investigadas. As mesmas foram obtidas através de informações prévias, constantes no prontuário eletrônico do paciente, considerando-se as respostas para o motivo da internação e as doenças crônicas registradas, e confirmadas por meio de entrevista telefônica, com vistas a subsidiar a classificação dos grupos com ou sem multimorbidade.

O questionário incluía características sociodemográficas, doenças crônicas referidas e questões sobre o encaminhamento na alta hospitalar: 1) retorno ao serviço especializado do próprio hospital na especialidade em que esteve internado- atenção secundária; 2) encaminhamento ao serviço especializado do próprio hospital na especialidade diferente da que esteve internado- atenção secundária 3) encaminhamento do tipo contrarreferência, do serviço especializado para o nível básico - atenção primária e questões sobre o encaminhamento e comparecimento (na entrevista 30 dias após a alta hospitalar): 1) encaminhamento do tipo referência, do nível básico para o serviço especializado; 2) comparecimento na atenção primária e 5) comparecimento na atenção secundária.

As características de utilização de serviços de saúde (agendamento de retorno ao hospital no pós-alta, encaminhamento para a unidade de saúde, comparecimento na unidade de saúde após a alta, comparecimento no especialista após a alta) foram obtidas através da entrevista telefônica.

A coleta de dados contou com três etapas: *1ª etapa:* acesso ao sistema próprio de informática do hospital para coleta de informações sobre internação: nome do paciente, motivo da internação (CID-primário), data da internação, data da alta, motivo da alta e setor de internamento, com vistas a elencar pacientes elegíveis. *2ª etapa:* acesso ao prontuário eletrônico do paciente elegível, onde foram coletadas informações, sobre as doenças crônicas e contato telefônico. 3ª etapa: entrevista com questionário estruturado, por meio de ligação telefônica, podendo ser direcionada ao próprio paciente, cuidador ou familiar. Todas as etapas foram realizadas por pesquisadores previamente treinados. Todos os pacientes elegíveis durante o período da pesquisa foram considerados a participar do estudo. O tempo médio da realização das entrevistas foi de 20 minutos.

### Análise dos Dados

#### Variável dependente

Considerou-se como variável dependente a ocorrência (presença/ausência) de multimorbidade. Esta variável foi criada a partir da análise das respostas do motivo da internação e presença de doenças crônicas. Os critérios de inclusão do grupo sem multimorbidade foram a presença de apenas uma ou nenhuma doença crônica, a presença de doenças ou condições agudas e doenças ou condições crônicas submetidas à cirurgia de reparo.

Foram incluídos no grupo com multimorbidade os indivíduos que apresentaram a coocorrência de duas ou mais condições crônicas^11^, sendo elas: hipertensão arterial sistêmica (HAS); diabetes mellitus (DM); acidente vascular cerebral (AVC); dislipidemia; doenças cardíacas: história de cateterismo ou revascularização, insuficiência cardíaca congestiva (ICC), infarto agudo do miocárdio, arritmia, cardiopatia, doença da artéria coronária e coronariopatia; doenças pulmonares: doença pulmonar obstrutiva crônica, enfisema pulmonar e bronquite; doenças autoimunes: esclerose lateral amniotrófica, esclerose múltipla, pênfigo vulgar, lupus eritematoso sistêmico; demências: alzheimer, parkinson; transtornos mentais: ansiedade e depressão; câncer; doença renal; hepatite alcoólica; epilepsia; dor crônica: cervicalgia, dor dorsal, dor em joelhos, dor de origem central; anemia crônica; doenças reumáticas: artrite, artrose, gota; osteoporose; infecção crônica: osteomielite, infecção na coluna, úlcera venosa; polineuropatia; alteração visual; incontinência: urinária e fecal e hiperplasia prostática.

#### Variáveis independentes

Considerou-se como variáveis independentes o encaminhamento e comparecimento aos serviços de atenção primária e secundária à saúde, considerando-se o período de 30 dias após a alta hospitalar.

#### Análise dos dados

Os resultados foram analisados descritivamente por meio de frequência absoluta e relativa. Para fins de delineamento do caminho percorrido por pacientes com e sem multimorbidade, considerando a rede pública formal de saúde, elaborou-se um fluxograma, onde foi considerado que o paciente percorreu o trajeto na APS quando encaminhado no momento da alta hospitalar ou no momento do retorno ao especialista. Já no que tange à atenção secundária à saúde (ASS), considerou-se o encaminhamento na alta hospitalar, ou durante a consulta do paciente na APS.

O fluxograma consiste em uma técnica de representação gráfica que utiliza símbolos previamente convencionados, permitindo a descrição clara e precisa de determinado fluxo ou de um processo, bem como sua análise e redesenho^21^.

#### Ética da Pesquisa

Após a identificação dos entrevistadores e da instituição afiliada, foram explicados os objetivos do estudo, meios e intermeios de coleta, análise e divulgação de resultados e obtido consentimento verbal dos participantes. Para evitar perda de informação, todos os participantes foram treinados a preencherem um formulário online durante a entrevista telefônica e como as questões eram fechadas, não houve a necessidade de gravação.

A pesquisa foi aprovada pelo Comitê de Ética em Pesquisas com seres humanos de uma Instituição de Ensino Superior (parecer nº 2.461.494/2018; CAAE: 81453417.1.0000.0105, respeitando os ditames da resolução 466/12 do Conselho Nacional de Saúde e a Declaração de Helsinki.

## Resultados

A amostra foi formada efetivamente por 445 pacientes internados, sendo 306 (68,76%) com ausência e 139 (31,23%) com presença de multimorbidade.

**Figura 1:**
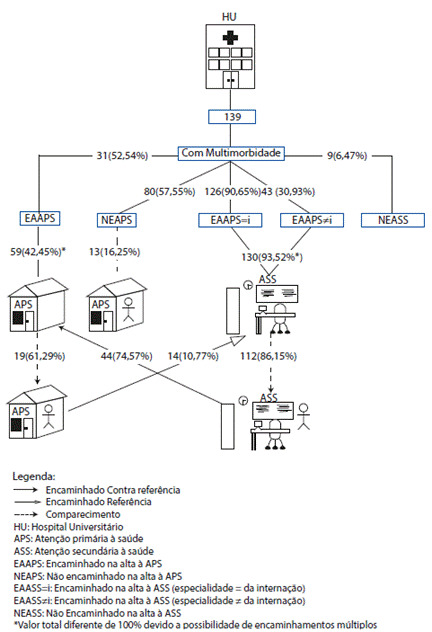
Caminho percorrido na rede pública formal de saúde por pacientes com multimorbidade, considerando-se o período de pós-alta hospitalar. Hospital Universitário. Paraná. Brasil, 2019.

Em relação ao grupo com multimorbidade (n=139) e a APS, 59 sujeitos (42,45%) relataram terem sido encaminhados a algum ponto da rede de APS após a alta hospitalar (n=31; 52,54%) ou após o retorno ao especialista do hospital (n=44; 74,57%). Alguns pacientes relataram também que o encaminhamento à APS ocorreu por iniciativa de mais de um profissional, considerando-se o momento da alta do hospital e da consulta de retorno com o especialista. Dos pacientes que foram encaminhados no momento da alta hospitalar, 19 (61,29%) efetivamente compareceram à consulta na APS. O comparecimento dos pacientes encaminhados no momento do retorno ao especialista não pode ser verificado, uma vez que a maioria afirmou que suas consultas de retorno ocorreram após 30 dias da alta hospitalar, período posterior ao término da coleta de dados desta pesquisa. Ainda, dos 80 pacientes (57,55%) que não foram encaminhados à atenção primária ou não souberam responder, 13 (16,25%) procuraram a APS no período investigado, mesmo sem encaminhamento, para tratar de questões relativas à sua internação (Figura 1).

A Figura 1 mostra também a relação do grupo com multimorbidade e a ASS: 130 (93,52%) entrevistados relataram terem sido encaminhados a algum ponto da rede de atenção secundária à saúde logo após a alta hospitalar, sendo 126 (90,65%) encaminhados para consulta na mesma especialidade que motivou a internação e 43 (30,93%) encaminhados a alguma outra especialidade médica. Ainda, 14 (10,77%) entrevistados afirmaram terem sido encaminhados à atenção secundária por profissionais da APS e 9 (6,47%) disseram não terem sido encaminhados ou não souberam responder. Alguns pacientes relataram também que o encaminhamento à ASS ocorreu por iniciativa de mais de um profissional, considerando-se a equipe de saúde do hospital e da atenção primária. Dentre a totalidade de pacientes encaminhados pela equipe do hospital, independentemente da destinação da especialidade médica, a maioria compareceu à consulta (n=112; 86,15%).

Fonte: A autora.

Da totalidade de pacientes com multimorbidade que estiveram internados, apenas 25 (17,98%) compareceram a todos os pontos da rede de saúde, considerando-se APS e ASS, em até trinta dias após a alta hospitalar.

**Figura 2:**
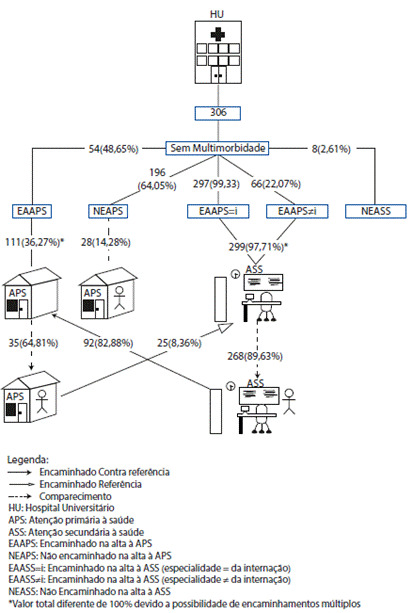
Caminho percorrido na rede pública formal de saúde por pacientes sem multimorbidade, considerando-se o período de pós-alta hospitalar. Hospital Universitário. Paraná. Brasil, 2019.

No que concerne ao grupo sem multimorbidade e a APS, 111 sujeitos (36,27%) relataram terem sido encaminhados a algum ponto da rede de APS após a alta hospitalar (n=54; 48,65%) ou após o retorno ao especialista do hospital (n=92; 82,88%). Alguns pacientes relataram também que o encaminhamento à APS ocorreu por iniciativa de mais de um profissional, considerando-se o momento da alta do hospital e da consulta de retorno com o especialista. Dos pacientes que foram encaminhados no momento da alta hospitalar, 35 (64,81%) efetivamente compareceram à consulta na APS. O comparecimento dos pacientes encaminhados no momento do retorno ao especialista não pode ser verificado, uma vez que a maioria afirmou que suas consultas de retorno ocorreram após 30 dias da alta hospitalar, período posterior ao término da coleta de dados desta pesquisa. Ainda, 196 pacientes (64,05%) que receberam alta hospitalar não foram encaminhados à atenção primária ou não souberam responder. Entretanto, mesmo sem encaminhamento, 28 (14,28%) procuraram a APS no período investigado, para tratar de questões relativas à sua internação (Figura 2).

Fonte: A autora.

A Figura 2, evidencia também a relação do grupo sem multimorbidade e a ASS: 299 (97,71%) entrevistados relataram terem sido encaminhados a algum ponto da rede de atenção secundaria à saúde logo após a alta hospitalar, sendo 297 (99,33%) encaminhados para consulta na mesma especialidade que motivou a internação e 66 (22,07%) encaminhados a alguma outra especialidade médica. Ainda, 25 (8,36%) entrevistados afirmaram terem sido encaminhados à atenção secundária por profissionais da APS e 8 (2,61%) disseram não terem sido encaminhados ou não souberam responder. Alguns pacientes relataram também que o encaminhamento à ASS ocorreu por iniciativa de mais de um profissional, considerando-se a equipe de saúde do hospital e da atenção primária. Dentre a totalidade de pacientes encaminhados pela equipe do hospital, independentemente da destinação da especialidade médica, a maioria compareceu à consulta (n=268; 89,63%).

Da totalidade de pacientes sem multimorbidade que estiveram internados, 67 (21,89%) compareceram a todos os pontos da rede de saúde, considerando-se APS e ASS, em até trinta dias após a alta hospitalar.

## Discussão

O fluxo da rede pública e formal de cuidado em saúde de pacientes hospitalizados evidenciou alto encaminhamento e comparecimento na ASS e um baixo encaminhamento e comparecimento na APS, para ambos os grupos investigados. Ainda, apenas 17,98% dos pacientes com multimorbidade e 21,89% dos sem multimorbidade foram atendidos nos três níveis de atenção. Entende-se que a semelhança de comparecimento em todos os pontos da rede por ambos os grupos é um problema, por tratar de forma igual populações desiguais e consequentemente com necessidades diversas, rompendo-se aqui o princípio doutrinário do SUS: a equidade.

Também o número de comparecimento na APS é extremamente baixo, para ambos os grupos, pois todos os pacientes deveriam passar por este ponto de atenção, configurando um retrato claro do funcionamento inadequado da RAS na área de abrangência desse estudo, e de falha no cumprimento do papel ordenador da rede e coordenador do cuidado pela APS. Para a APS cumprir o papel de ordenadora do sistema de atenção à saúde, na perspectiva da RAS, deve cumprir três funções essenciais: função resolutiva de atender 85% dos problemas mais comuns de saúde, função coordenadora dos fluxos e contrafluxos de pessoas, produtos e informações nas redes e função de responsabilização pela saúde da população usuária adscrita a sua região^22^.

Em relação ao baixo número de encaminhamentos à APS e ao seu papel central na RAS, é importante ressaltar que a responsabilidade desse nível de atenção é a promoção de saúde, o tratamento, a reabilitação e a redução de danos à saúde. Assim, visto que a totalidade da população investigada experienciou uma internação hospitalar, e tendo ciência de que o pósalta é um período de vulnerabilidade a complicações à saúde especialmente nesses pacientes, seria esperado que os mesmos tivessem acompanhamento de saúde pela APS, de modo a garantir a longitudinalidade e a integralidade de seu cuidado. A RAS preconiza o papel central na APS, de modo articulado com os demais pontos de atenção da rede, destacando a necessidade de uma atuação forte e robusta, com boa cobertura populacional, como item essencial para a constituição da rede de atenção às pessoas com doenças crônicas^15^.

Ainda, apesar da frequência de pacientes a todos os níveis de atenção não ser condição *sinequa non* no cuidado à saúde, 93,52% e 97,71% dos pacientes com e sem multimorbidade, respectivamente, foram encaminhados à ASS e apenas menos da metade deles foram encaminhados à APS, o que denota uma falta de vínculo destes sujeitos com a APS e falha do hospital no contrarreferenciamento. Ademais, pode-se ponderar também que ocorre falta de valorização/reconhecimento, por parte da atenção terciária, de que a APS é a ordenadora da rede e coordenadora do cuidado, sendo o contrarreferenciamento do paciente a ela fundamental para garantia do cuidado integral. Neste contexto é fundamental a comunicação entre os diferentes níveis de atenção, requerendo, deste modo, sistemas de informação em saúde eficientes e comunicantes, condição ainda incipiente no município investigado.

No que diz respeito aos pacientes com multimorbidade, a literatura reforça a importância do acompanhamento destes pacientes pela APS, uma vez que grande parte das morbidades mais frequentes são sensíveis à atenção primária^23^. Outro aspecto relevante relacionado a este nível de atenção é o benefício do acompanhamento regular de pacientes com multimorbidade por médicos generalistas, que devido potencial de garantia de continuidade do cuidado, parece ter um papel fundamental na melhora de desfechos negativos, como hospitalização, múltiplas hospitalizações e readmissões,^9^^,^^11^.

Apesar de consolidada a melhoria da condição de saúde do paciente com multimorbidade atendido na APS, é importante investigar a causa da ausência de encaminhamento destes pacientes ao nível primário. Pode-se inferir que o pequeno número de pacientes com multimorbidade acompanhadas pela APS decorra da fragmentação do cuidado, demonstrando uma falta de comunicação e de referenciamento entre os diferentes níveis da RAS, bem como a deficiência na tarefa de coordenação do cuidado pela APS. O fato pode ainda decorrer de lacunas do próprio sistema de saúde, que levam os usuários a buscarem os serviços de maior complexidade como porta de entrada da rede de saúde, por motivo de dificuldade de acesso à rede primária ou ao estigma que os hospitais são mais resolutivos^24^.

Outro aspecto observado foi a tendência discreta de maior número de encaminhamentos à APS no grupo com multimorbidade, que pode ser explicada pela maior predisposição à complicações^25^, maior necessidade de orientação, apoio e cuidado familiar, devido à complexidade da interação de múltiplas doenças e tratamentos.

Mundialmente, pacientes com múltiplas doenças vêm determinando quadros habituais na rotina dos serviços primários de saúde, sendo relevantes e atuais as reflexões sobre intervenções efetivas para a melhoria dos desfechos em saúde desses sujeitos não somente no âmbito da atenção primária, mas também sobre os seus desdobramentos no contexto da comunidade e dos domicílios. Assim, o repensar das práticas primárias à saúde de pacientes multimórbidos deve perpassar não somente pela instrumentalização e sensibilização de profissionais; mas também de familiares, nos quais as dimensões afetivas, cognoscentes e de cuidado devem estar presentes.

Em pacientes com doenças crônicas agudizadas, a internação hospitalar tem grande chance de acontecer e pode se tornar mais complicada com o aumento de morbidades, resultando em maior tempo de permanência, maior mortalidade, maior probabilidade de internações múltiplas e readmissões^11^ e maior gasto com serviços de saúde^26^. Nesse sentido, as hospitalizações deveriam ser eficientemente utilizadas, reforçando o manejo adequado da multimorbidade na APS e incluindo o domicílio como ambiente terapêutico^8^. Porém, quando necessária a internação hospitalar, torna-se fundamental a integração entre os serviços de saúde, com compartilhamento de informações^11^.

Verificando-se agora o comparecimento na APS e na ASS, pode-se examinar maior frequência, de ambos os grupos investigados, na ASS. Atribui-se este achado à falta de valorização da APS, acredita-se que a desvalorização ocorre não somente por parte da população que não está comparecendo neste serviço mesmo quando encaminhada, mas também por parte da própria equipe de saúde, ao frequentemente não considerar esse serviço durante o seu processo de encaminhamento. Starfield^27^ relata que a grande maioria de pacientes atendidos por especialistas não é reencaminhada ao médico responsável pelo primeiro encaminhamento.

A desvalorização da APS, bem como o maior encaminhamento à ASS em detrimento da APS pode ser explicado pelo paradigma vigente da racionalidade biomédica, que sedimentou a atenção à saúde, fragmentando-a em inúmeros especialistas e partes restritas do corpo humano, condição que está arraigada na cultura dos usuários do sistema, que valorizam serviços especializados em detrimento a APS, assim como na dos profissionais que atuam no sistema. Especialmente os multimórbidos, são tratados por diversas especialidades distintas, com variadas intervenções como se fossem pacientes diferentes, em que com frequência ninguém se responsabiliza pela globalidade do tratamento e cuidado, dificultando uma atenção integral^28^.

Apesar do reconhecimento atual da importância de superação da fragmentação em saúde, a maioria das universidades ainda formam profissionais de forma fragmentada, através de um modelo pedagógico conteudista e segmentado^12^, acredita-se que a consequente inadequação dos recursos humanos resvale na organização dos serviços em saúde, que não obstante em teoria ajam de forma integral, na prática ainda não conseguem alcançar a integralidade, como exemplo a ser citado o baixo encaminhamento e comparecimento na APS no presente estudo.

Ainda em relação à ASS e a diferença entre os grupos, observa-se uma menor solicitação de retorno ao hospital em pacientes com multimorbidade, considerando-se a mesma especialidade que motivou a internação (90,65% e 99,33%, para pacientes com e sem multimorbidade, respectivamente). Este fato pode estar relacionado à maior prevalência de pacientes em situação de pós-operatório no grupo sem multimorbidade, uma vez que é rotina usual entre profissionais da saúde solicitar o retorno de pacientes recém-operados ao hospital.

Quando analisado o encaminhamento a outro especialista, não relacionado à especialidade que motivou a internação, no momento da alta hospitalar (30,93% e 22,07%, para pacientes com e sem multimorbidade, respectivamente), nota-se um número maior de encaminhamento no grupo com multimorbidade, em comparação ao grupo sem multimorbidade. O maior encaminhamento dos pacientes multimórbidos é esperado, visto que os especialistas têm um papel importante no cuidado de doentes complexos e seu uso aumenta com a frequência de condições crônicas, onde um grande número de especialistas pode ser esperado^11^.

No entanto, a literatura expõe, em caso de pacientes com multimorbidade, que direcionar o cuidado a doenças isoladas possa subutilizar as possibilidades de melhora ou até levar a prejuízos à saúde^3^^,^^23^, tais como polifarmácia, decorrente da falha de comunicação entre diferentes profissionais^3^ ou do senso comum de que cada especialidade médica pressupõe o uso de medicamentos distintos e maior número de internações hospitalares, atribuindo-se como causa o fato de especialistas tratarem estes pacientes de forma fragmentada e descoordenada^9^.

Nesse sentido, recomenda-se o uso racional de especialistas em pacientes com multimorbidade, reiterando a relevância da nomeação de um médico responsável para gerir o cuidado de pacientes complexos^29^ uma vez que a consulta com diversos especialistas dificulta a continuidade do cuidado, fato que parece ter um papel fundamental para melhorar os desfechos^11^^,^^27^. Outro aspecto de fundamental importância é a comunicação clara entre os profissionais, uma vez que muitas prescrições são realizadas inicialmente por especialistas e repetidas na APS.

Frente a este contexto, o bom funcionamento da RAS depende do compartilhamento entre os profissionais dos diferentes níveis de atenção à saúde. Os pontos de atenção especializada e hospitalar no cuidado às pessoas com doenças crônicas devem ser complementares e integrados à atenção primária, superando a fragmentação comumente detectada nos serviços de saúde brasileiros. A maneira mais eficiente de relação entre APS e ASS é a coordenação do cuidado com responsabilidade solidária entre os profissionais sendo, para tanto, essencial estabelecer responsabilidades e garantir comunicação e transferência segura do cuidado, através do compartilhamento e discussão de planos de cuidados entre os diferentes níveis de atenção^15^.

Nessa conjuntura, diretrizes clínicas, estruturadas a partir da construção de uma linha de cuidado, que considerem a vulnerabilidade de pacientes portadores de grupos de doenças crônicas tornam-se essenciais para o cuidado à saúde longitudinal e para a organização dos serviços públicos de saúde.

Diante deste cenário, é relevante o uso eficiente do sistema de saúde, através do fortalecimento e execução de políticas públicas de saúde vigentes, como Política Nacional da Atenção básica (PNAB)^30^, Política Nacional de Atenção Hospitalar (PNHOSP)^31^e Política Nacional da Humanização (PNH)^32^.

A PNAB foi reestruturada para atuar dentro da lógica de rede, como ordenadora do cuidado, garantindo o ordenamento do cuidado, a integralidade e o cuidado longitudinal^30^, tão importantes para o paciente multimórbido. A PNHOSP deve ser implantada em todo âmbito hospitalar e traz como princípio que todo o enfoque do cuidado seja na atenção primária, dentro de uma lógica de rede^31^, fato que não ainda está acontecendo no município investigado.

Outra política importante a ser destacada é a PNH, que fala da importância da integralidade do cuidado, do acolhimento baseado em critérios de risco e do encaminhamento de pacientes onde for necessário dentro da estrutura de rede, garantindo a integralidade e o atendimento às demandas e necessidades^32^. Essas três políticas são exemplos que endossam o cuidado centrado no paciente, ordenado por uma rede de atenção à saúde, portanto, é essencial sair da teoria e colocar em prática estas políticas.

Acredita-se que a fragmentação do cuidado observada neste estudo decorra da falha na comunicação entre os diferentes níveis de atenção, uma vez que a atenção primária precisa ter conhecimento do momento que o paciente deixa a atenção terciária para que possa gerenciar o cuidado de forma eficiente. As RAS vieram com o intuito de suprir a fragmentação do cuidado, de modo que a APS conseguisse atender de forma integral os pacientes^33^, no entanto, desde a publicação da RAS, já se passaram dez anos da reorganização de funcionamento do SUS e contudo o trabalho evidenciou que o município investigado ainda não consegue colocar em prática o que se idealizou para a atenção aos pacientes, de forma eficiente, devido a falha na comunicação entre os níveis de atenção.

Diante deste cenário, precisa-se capacitar as equipes de saúde dos três níveis de atenção, acerca do papel da APS como ordenadora do cuidado. Todos os níveis de atenção necessitam valorizar e enxergar o protagonismo da APS nesse processo e ainda se faz necessário que a própria APS se empodere para tal. Todavia é imprescindível que a APS acompanhe o caminho percorrido na rede de saúde pelos pacientes para que possa acompanhar o cuidado em saúde, garantindo a longitudinalidade do cuidado e ordenação da rede. Nesse âmbito, o desenvolvimento de um sistema de informação eficiente é indispensável para a ordenação na rede pela APS. No município observado, não existe um sistema de informação comum entre os diferentes pontos da rede, não sendo possível a comunicação entre os diversos pontos da rede de saúde.

Outro ponto importante, além da capacitação das equipes, é o número suficiente de recursos humanos na APS, para que a mesma consiga exercer seu papel de ordenadora do cuidado. A cobertura da atenção primária no município pesquisado, no período da coleta de dados, foi de apenas 79%^34^, a baixa cobertura demonstra uma sobrecarga das equipes para suprir o básico, ficando restritas ao assistencialismo em detrimento da gestão do cuidado continuado.

Este estudo apresenta limitações que devem ser ponderadas. Os achados deste estudo devem ser interpretados com cautela, uma vez que estudos transversais não permitem a verificação de fatores causais^35^^,^^36^, sendo importante o desenvolvimento de estudos longitudinais.

Por ser um trabalho de base hospitalar, seus achados não podem ser extrapolados para a população geral, pois a internação aumenta as chances de diagnósticos de doenças^8^, e exclui pacientes sem acesso à rede de serviços de saúde. A classificação de multimorbidade foi realizada através da contagem de condições ou agravos de saúde, e não levou em consideração a severidade das doenças. Entretanto esta é a forma mais comum de classificação em estudos de base populacional como este^37^. Outras limitações poderiam ser atribuídas à construção do fluxograma da rede de cuidado considerar apenas o período de trinta dias após a alta hospitalar, autorrelatos e o viés da memória.

Contudo, esses limites não invalidam ou diminuem a relevância e a singularidade da proposição do presente estudo, uma vez que não foi encontrado na literatura trabalho que discuta o caminho percorrido na rede de saúde e a multimorbidade em uma proposta inversa (contrareferenciamento) do comumente encontrado, o paciente saindo da atenção terciária para a APS, reforçando a necessidade de estudos futuros, com informações longitudinais, que diferenciem internações clínicas de cirúrgicas e ampliem o período de avaliação após o período de alta.

Uma potencialidade deste estudo é o uso simultâneo de diagnósticos médicos autorreferidos e registrados em prontuários, uma vez que a maioria dos estudos na área utilizam apenas diagnósticos autorreferidos, podendo incorrer em discordância com a realidade^35^.

## Conclusões

O acompanhamento de 30 dias após a alta hospitalar de pacientes com e sem multimorbidade possibilitou verificar uma alta prevalência de encaminhamento e comparecimento na ASS, um baixo encaminhamento e comparecimento na APS, e um baixo comparecimento a todos os pontos da rede de saúde, para ambos os grupos investigados.

Obaixo comparecimento a todos os pontos da rede de saúde, para ambos os grupos investigados, configuram um retrato do funcionamento inadequado da RAS na área de abrangência deste estudo e sinaliza a importância de maior incentivo ao acompanhamento de pacientes com multimorbidade na rede básica de saúde, especialmente no período de pós-alta, e a necessidade de integração dos pontos da rede de saúde brasileira, de modo a garantir a continuidade e integralidade do cuidado a estes pacientes.

Nesse sentido, acredita-se na necessidade de suficiência de recursos humanos na APS, capacitação permanentemente dos profissionais de saúde dos diferentes níveis de atenção e o fortalecimento do sistema de informação em saúde.

Dado o impacto social e o elevado custo que a multimorbidade representa ao sistema de saúde, novos estudos que avaliem o caminho percorrido na rede de saúde em pacientes que estiveram internados com multimorbidade se fazem necessários, a fim de aprofundar o tema.
